# Oral Presentations - Abstracts of the 16^th^ REDLARA Taller General, Medellin - Colombia, 27-30 April 2023

**DOI:** 10.5935/1518-0557.20230019

**Published:** 2023

**O-01. Is it all about the egg’s age? Euploid embryo transfer reproductive outcomes in autologous and egg donation cycles**

**Mauricio B. Chehin^1^, José Roberto Alegretti^2^, Thais S. Domingues^1^, Claudia Gomes^1^, João Pedro Junqueira Caetano^3^, Mariana Nicolielo^2^, Renata Erberelli^2^, Aline R. Lorenzon^4^**

^1^Clinical Department - Huntington Medicina Reprodutiva - Eugin Group, São Paulo, SP, Brazil

^2^Embryology Department - Huntington Medicina Reprodutiva - Eugin Group, São Paulo, SP, Brazil

^3^Clinical Department - Huntington Pró-Criar Medicina Reprodutiva - Eugin Group, São Paulo, SP, Brazil

^4^Scientific Coordinator - Huntington Medicina Reprodutiva - Eugin Group, São Paulo, SP, Brazil

**Introduction: Decline in reproductive potential is intrinsically related to aging. In women, diminished ovarian reserve and oocyte quality is more prominent after 35 years old. In the last decades, the increase of maternal age in ART (assisted reproductive technology) centers expanded the number of oocyte donation cycles, with the expectation of surpass oocyte aging and the decrease in ovarian reserve and proving an equivalent pregnancy rates as younger patients. The information that women until their mid-40s that undergo an IVF treatment with egg donation are able to maintain the same live birth rates as younger patients using their own oocytes is widely disseminated. The aim of this study was to compare the reproductive outcomes of euploid embryo transfers in patients undergoing autologous versus donor cycles.**

**Materials and Methods: This is a retrospective cohort study with patients undergoing IVF (in vitro fertilization) cycles with preimplantation genetic testing for aneuploidies (PGT-A) by Next Generation Sequencing (NGS) technology according to medical referral between Jan/2016 and Jul/2020. Positive pregnancy test (PP), clinical miscarriage (CM) and live birth (LB) rates were compared between patients using autologous or donated oocytes. Fisher test were apply for statistical analysis and** *p***<0.05 was considered significant.**

**Results: In total, 3189 patients - 2860 patients using autologous oocytes (89.7%) and 329 patients using donated oocytes (10.3%) were included in this cohort. There were 3573 embryo transfers - 3203 autologous and 370 from donated oocytes - with 4764 biopsied blastocysts - 4264 autologous and 500 from donated oocytes - in a mean of 1.33 and 1.35 blastocysts per transfer in autologous and donated cycles respectively. Mean maternal age in autologous cycles was 37.6±4.3 years old and 43.4±4.0 for donated cycles. The mean age of oocyte donors in our program is 24.99±3.86 years old. Positive pregnancy test and live birth rates were higher in autologous (PP: 59.3% and LB: 39.2%) compared to donated cycles (PP: 53.8% and LB: 32.4%,** *p***=0.039 and 0.011 respectively). Miscarriage rates were higher in donated cycles (15.6%** *vs***. 10.6%,** *p***=0.042).**

**Discussion: The live birth rates between autologous and donated cycles were statistically different in out cohort. This highlights the need of attention when advertising to reproductive advanced maternal age patients on their chance of success when considering undergoing a treatment with egg donation. An important factor that is often dismissed is the uterine physiology. The endometrial tissue is susceptible to aging. Recently data on this subject has shown that the mostly affected pathways related to aging in the endometrium are cellular remodeling, cell motility and migration, and the immune response (Loid et al., 2022). All of them are crucial for embryo implantation and the development of early pregnancy. Furthermore, in pregnancies with oocyte donation, the fetus is completely allogenic to the maternal organism, which also affects the immunological recognition and tolerance during gestation. This is a large cohort study, including only euploid embryos, which excludes any bias on ploidy status when comparing autologous versus donated oocytes cycles. A limitation of our study is that we have not considered paternal age in this analysis.**

**Conclusion: Establishment of pregnancy does not depend only on the age of the oocyte. Other important variables that are related to maternal aging such as endometrial tissue physiology, health comorbidities and the effect of immune response should be taken into consideration when counseling reproductive advanced age patients in ART centers.**

**Reference**

Loid M, Obukhova D, Derks K, Meltsov A, Kask K, Altmäe S, Saare M, Peters M, Esteki MZ, Salumets A. Does endometrium age? The endometrial transcriptome of advanced reproductive age patients reveals the signs of cellular ageing, altered immune response and compromised receptivity. Hum Reprod. 2002;37:deac104.074.

**O-02. Management of patients with endometriosis and infertility: laparoscopy treatment and spontaneous pregnancy rate**

**Yanina E Rodríguez^1^, Lautaro Tessari^1^, Florencia Perotti^1^, Marcela Irigoyen^1^, Antonio Cattaneo^1^, Esteban Grasso^2^, Rosanna Ramhorst^2^, Diego Gnocchi^1^**

^1^ Fertilis Medicina Reproductiva, Buenos Aires, Argentina

^2^ CONICET. Universidad de Buenos Aires. Instituto de Química Biológica de la Facultad de Ciencias Exactas y Naturales (IQUIBICEN). Buenos Aires, Argentina

**Introduction: Endometriosis is a frequent worldwide disease, with a prevalence estimate between 2%-10% for women in the general, and increases up to 50% in infertile women. The high efficacy of modern-day assisted reproductive technology (ART) has led to progressively adopting ART-first approaches, particularly for women with endometriosis. These characteristics of infertility management lead to the following questions: Does laparoscopy play a role in these patients? What other factors are involved for a spontaneous pregnancy? Here we propose to evaluate the spontaneous pregnancy rate after surgical treatment in patients with endometriosis.**

**Materials and Methods: Retrospective study, from 2014 to 2020 of 2 years followup of patients with endometriosis-related infertility and laparoscopic surgery from “Fertilis -Sanatorio las Lomas”. Most of the patients reported symptoms improvement. Of 303 after surgery, 200 complied with selection criteria and were analyzed and classified according to fertility treatment as: patients that used ART immediately 16.5% (ART-p); while 83.5% waited for an spontaneous pregnancy. From the last group 71.9% reach a spontaneous pregnancy (SP-p) while 28.1% was not able to and required ART (23.5%, NSP-p). Groups have no significant demographic differences. Age, endometriosis ASRM score (EAS), initial symptoms, primary/secondary sterility, time of sterility, tubal and uterine quality, and time until pregnancy were recorded. Age was also grouped as <30, 30-34, 35-39 and 39+ years. Data was analyzed with GraphPadPrism using chi-square, ANOVA and T-test. The decision tree was made using RPart-package on R.**

**Results: After laparoscopic treatment 71.9% of patients that waited for a spontaneous pregnancy achieved it. Patients that choose ART increased with the age, especially in the 39+ years group as other factors not related with endometriosis are involved such as ovarian reserve. We observed that ART-p shown a tendency of higher EAS compared with patients that waited for a spontaneous pregnancy. We then focus our analysis into SP-p** *vs***. NSP-p groups. We did not observe significant differences in the EAS though there is a higher proportion of patients with lower EAS(I) between SP (21.2%) and NSP patients (11.4%). Of all other studied variables, only tubal quality showed a significant difference, with a higher percentage of regular quality on the NSP-p (22.8%** *vs***. 8.5%,** *p***<0.05). When we studied how long it took to achieve pregnancy, we observed that NSP-p took almost 1.8 times more than SP-p (10.2±3.7** *vs***. 5.7±3.6 months,** *p***<0.001). Interestingly, if we only consider the time since the change from waiting for spontaneous pregnancy to ART, the patients achieve the pregnancy in a similar time that the SP ones (4.9±3.7 months), suggesting that the NSP patients could have benefited if they were identified early. As none of the studied variables by themselves were able to identify these patients, we performed a multivariable approach. Using R and we obtained a decision tree with 79.6% accuracy and 53.3% sensitivity.**

**Discussion, Conclusion: Our data are consistent with previous clinical studies regarding the management options of endometriosis-related infertility, including an increase in spontaneous pregnancy after surgical treatment. Although we didn’t find a clear correlation between EAS and spontaneous pregnancy, a tendency between lower score and better outcome is present. We also found a group of patients that were not able to get pregnant spontaneously and required ART. To identify these patients at the clinic, we develop a decision tree algorithm, easy to follow, that has 79.6% accuracy and 53.3% sensitivity. Further studies could improve the decision tree found. The early identification of these patients will decrease the time between surgery and pregnancy, improving the general outcome.**

**O-03. Presence of Candida species in human endometrium: something to pay attention to?**

**Marta Pérez-Sánchez^1^, Antonio Martínez-Lara^2^, Claudia Díaz^1^, David Cotán^2^, Rosa Gómez^1^, Carmen Morales^1^, Ana Escrivá^1^, Jose Antonio Horcajadas^3^**

^1^Laboratory, Pronacera Therapeutics S.L, Seville, Spain

^2^Research, Pronacera Therapeutics S.L., Seville, Spain

^3^Research, Sinae S.L., Seville, Spain

**Introduction: Formerly, the human uterine cavity was considered a sterile section. However, in recent years several studies have refuted this dogma. A uterine microbiome has been detected that seems to have a protective role against infections, and to modulate the immune cells necessary for embryo implantation. However, the presence of pathogenic or potentially pathogenic microorganisms in endometria has been related to impaired fertility. For instance, the presence of certain species such as Candida spp. has been related to endometrial conditions such as endometriosis and intrauterine adhesions, however its influence on endometrial receptivity and functionality has not been evaluated until now.**

**Material and Methods: A total of 74 patients (average age = 39.29 years) were included in the study. Patients went through an endometrial biopsy for microbiome testing. RT-qPCR was performed for detection of certain microorganisms and pathogens. Samples were selected based on the absence of pathogens and classified according to the presence or absence of any** *Candida* **species: 13 samples with presence of almost one** *Candida* **specie (average age = 40.46 years) and 61 samples with absence of any** *Candida* **specie (average age = 39.04 years). Then, a gene expression analysis of a 182 genes-panel related to endometrial functionality and receptivity was performed by microfluidic RT-qPCR in the endometrial samples. Data was analyzed by Delta-Delta Ct method, and Fold change was determined using Wilcoxon test.**

**Results: 16 out of 182 analyzed genes (8.79%) show significant differences (***p***<0.05) between groups (***Candida presence vs. Candida absence***). Particularly, 5 of them (AREG, CXCL6, CCR7, IL1B and IL8) are overexpressed in samples with** *Candida* **presence whereas the remaining 11 (ALDH1A3, CAPN6, CTNNA2, CRISP3, IL1R1, PRL, DKK1, MMP26, PROK1, LRRC17 and PTPRZ1) are overexpressed in samples without** *Candida* **presence.**

**Discussion: The present study shows the potential influence of Candida species in the expression profile of a set of genes closely related to endometrial receptivity and functionality. Genes overexpressed in samples with** *Candida* **presence are related to endometrial receptivity and immune response such as interleukins, chemokines, and chemokine receptors families. Furthermore, samples without** *Candida* **species present an overexpression of genes related to pathways of endometrial receptivity and early pregnancy development, which include regulation of endometrial cell proliferation and decidualization. However, further studies will be required to determine the clinical significance of** *Candida* **presence for reproductive success. In addition, it would be necessary to evaluate if the presence of extra pathogens could alter these observations.**

**Conclusion: Presence of** *Candida* **species in human endometrium affects the expression profile of genes related to endometrial receptivity and functionality with uncertain clinical significance.**

**References**

Benner M, Ferwerda G, Joosten I, van der Molen RG. How uterine microbiota might be responsible for a receptive, fertile endometrium. Hum Reprod Update. 2018;24:393-415.

Gazvani R, Fowler PA, Coyne L, Odds FC, Gow NAR. Does Candida Albicans Play a Role in the Etiology of Endometriosis? J Endometr Pelvic Pain Disord. 2013;5:2-9.

Moreno I, Garcia-Grau I, Perez-Villaroya D, Gonzalez-Monfort M, Bahçeci M, Barrionuevo MJ, Taguchi S, Puente E, Dimattina M, Lim MW, Meneghini G, Aubuchon M, Leondires M, Izquierdo A, Perez-Olgiati M, Chavez A, Seethram K, Bau D, Gomez C, Valbuena D, et al. Endometrial microbiota composition is associated with reproductive outcome in infertile patients. Microbiome. 2022;10:1.

Zhao X, Sun D, Zhang A, Huang H, Li Y, Xu D. Candida albicans-induced activation of the TGF-β/Smad pathway and upregulation of IL-6 may contribute to intrauterine adhesion. Sci Rep. 2023;13:579.

**Table 1:** Results.

Parameter	Young Group (< 37 years)	APA Group (≥ 37 years)	*p*-value
Male Age (years)	32.8±3.3	42.5±4.3	< 0.001
Oocyte Donor Age (years)	23.1±2.5	21.5±2.1	> 0.05
Female Receptor Age (years)	41.0 ± 5.3	40.7±6.3	> 0.05
Semen Volume (mL)	3.1±1.4	2.8±1.9	> 0.5
Sperm Concentration (x106/mL)	59.6±21.2	49.1±30.6	> 0.5
Progressive Sperm Motility (%)	57.3±12.7	58.2±11.9	> 0.5
Normal Sperm Morphology (%)	3.4±3.4	3.5±2.7	> 0.5
Sperm DNA Fragmentation (%)	3.3±3.4	5.4±3.9	> 0.5
Inseminated Oocytes	15.0±4.8	14.4±4.2	> 0.5
Fertilization Rate (%)	86±11.3	84±12.5	> 0.5
Cleavage Rate (%)	90±9.9	94±5.8	> 0.5
Blastocyst Rate (%)	58±56	56±22	> 0.5
Pregnancy Rate (%)	50	50	> 0.5
Miscarriage Rate (%)	12	26	< 0.001

**O-4. Paternal age over 37 years increases the probability of miscarriages in an oocyte donation program**

**Conrado Avendaño^1^, Vilma Lombardo^1^, Gustavo Manavella^1^, Fany Villalba^1^, Nathalia Toñanez^1^, Óscar Ruíz Valdéz^1^, Rodrigo Britos^1^, Carlos Roger Molinas^1^**

^1^Neolife Medicina y Cirugía Reproductiva. Asunción, Paraguay

**Introduction: The trend of delaying childbearing has led to an increase in the average age of both mothers and fathers at the time of conception. While the impact of advanced maternal age on reproductive outcomes has been widely studied, there is still ongoing discussion around the influence of advanced paternal age (APA) on reproductive outcomes. Preliminary research suggests that males between the ages of 35 to 40 may experience a decline in semen quality, which can negatively impact reproductive outcomes. This study aimed to evaluate the effect of APA on assisted reproductive treatment in an oocyte donation program.**

**Material and Methods: This retrospective study enrolled infertile couples who participated in our oocyte donation program. We have excluded cryopreserved semen, sperm from testicular biopsy, and patients with azoospermia or cryptozoospermia. Healthy female donors under 30 years old, without pre-existing pathologies, provided anonymous gamete donation. All procedures were performed in a fresh cycle using non-vitrified oocytes and embryos. Patients aged 37 years and above were classified as the APA group, consistent with our prior research. The study included 111 couples, and the evaluation focused on fertility rate, cleavage rate, blastocyst rate, pregnancy outcomes, and first-trimester miscarriage. Data were presented as median ± standard deviation (SD) or as a percentage of the total. Mann-Whitney (U-test) and Chi-squared tests were applied to compare variables between the study groups. Statistical significance was considered when** *p***<0.05.**

**Results: All results are shown in the table. Our study showed no significant differences in fertilization rate, embryo development, or pregnancy rate between the APA and younger groups. However, a significant increase in miscarriage was observed in the APA group compared to the younger group.**

**Conclusion: In recent years, there has been a steady increase in parental age. In the context of an oocyte donation program, we examined the influence of APA and found that it may not have an impact on the first step of assisted reproductive outcomes, such as fertilization rate, embryo development, or pregnancy rate. Nevertheless, our results indicate that APA may increase the likelihood of natural miscarriage. As a result, we suggest that individuals seeking to conceive should be advised that semen samples from men over the age of 37 may pose a greater risk of pregnancy loss, and that APA should be a relevant consideration in pre-conception counseling. This study provides valuable insights into the impact of APA on assisted reproductive treatment in an oocyte donation program and highlights the need for further research in this area. Larger-scale studies are needed to validate these findings and further explore the impact of APA on reproductive outcomes.**

**O-6. Relationship between serum progesterone levels, pregnancy and live birth outcomes. Experience of a Uruguayan center**

**Tamara Conde Rodrigues^1^, Magdalena Decia^1^, Gabriel De la Fuente^1^, Dana Kimelman**

^1^Clínica CREA, Montevideo, Uruguay

**Introduction:** The use of frozen embryo transfer (FET) in assisted reproduction has increased over the past years as a result of improvements in cryopreservation techniques. FET in a natural cycle has shown similar pregnancy rates than an artificial cycle with less obstetric complications which is why it has become the gold standard in our clinic. However patients in amenorrhea or irregular cycles still need to go through medicated cycles. In artificial cycles the addition of progesterone is a crucial step to achieve synchrony between the endometrium and the embryo´s development stage. Several International studies have address this topic showing significantly lower CPR when the progesterone level was < 9 on the day of the transfer. In the search for optimization and individualization of progesterone supplementation, our research question is if serum progesterone concentration on the day of the FET affects pregnancy and live birth rates in our population.

**Material and Methods:** A retrospective, single-center study was carried out in FET cycles performed between April 2019 and July 2021. Cycles included were FET of blastocyst stage embryos in patients who received an endometrial preparation with vaginal progesterone (800mg daily). All measurements of serum progesterone were performed on the day of the embryo transfer at the same center using the Roche Cobas e 411, 3^rd^ generation method. Patients with natural cycle endometrial preparation, who used donated embryos, or who went through preimplantation genetic testing were excluded. Patients were classified into two groups according to the progesterone level on the day of the FET, <9 and ≥9 ng/mL. The primary outcome was clinical pregnancy rates (CPR) and the secondary outcome was live birth rates (LBR). A p-value <0.05 was considered.

**Results: 115 cycles were included. The mean age of patients was 37.9 years (range 19-48 years), 41.2% of the cases corresponded to egg donation (OD) and 58.8% used their gametes. 74.6% of cycles were single embryo transfers and 25.4% were double embryo transfers. All embryos were transferred at a blastocyst stage (day 5 or day 6) and vitrification was the only cryopreservation method. Pregnancy rates in the group with a serum progesterone <9 ng/mL were 45.03%, while in the group ≥9 ng/mL PR was 51.2%, with no significant difference. LBR in the group with a serum progesterone <9 ng/mL was 35.05% while in the group ≥9 ng/mL was 35,21%, with no significant difference. The difference in PR and LBR were still not considered significant after controlling for possible confounders (age and procedure of the gametes).**

**Discussion and Conclusions: We observed a slight trend of better results in pregnancy rates and live birth rates with serum progesterone ≥9 ng/mL. However, the differences in CPR and LBR were not significant. Our results are similar to what has been published in the literature. The strength of our study is that to the best of our knowledge, it is the first study to examine data from an Uruguayan center which will help us standardize our FET techniques. Further research is needed in order to evaluate the optimal serum progesterone level on the day of frozen embryo transfer.**

**O-05. Progesterone-primed cycles result in slower embryos without compromising implantation potential and with the advantages of oral administration and potential cost reduction: A time-lapse imaging study**

**Edson Borges Jr.^1,2,3^, Daniela Paes de Almeida Ferreira Braga^1,2^, Amanda Setti^1,2^, Edward Carrilho^3^, Patrícia Guilherme^4^, Assumpto Iaconelli Jr.^1,2,3^**

^1^Scientifc Research Department - Fertility Medical Group, São Paulo, SP, Brazil

^2^Scientifc Research Department - Sapientiae Institute - Centro de Estudos e Pesquisa em Reprodução Humana Assistida, São Paulo, SP, Brazil. Zip: 04503-040

^3^Clinical Department - Fertility Medical Group, São Paulo, SP, Brazil

^4^IVF Laboratory - Fertility Medical Group, São Paulo, SP, Brazil

**Introduction: Analogs of GnRH have been used for decades to prevent the LH surge and premature ovulation. Improvements in cryopreservation have allowed for the option to break away from the standard sequence of stimulation-retrieval-transfer and to consider new strategies for pharmacological control of follicle growth. Considering this, progesterone/progestins have been included in ovarian stimulation protocols as an alternative to analogs of GnRH, with the advantages of being an oral treatment and providing more control over LH serum levels. Nevertheless, specialists have expressed concern over prolonged exposure of the developing follicles to progesterone. The goal of the present study was to investigate the impact of the use of progesterone on embryo morphokinetics and on the outcomes of intracytoplasmic sperm injection (ICSI) cycles.**

**Material and Methods: This cohort study, performed in a private university-affiliated IVF center from March 2019 - March 2021, included 236 freeze-all ICSI cycles and 2,768 injected oocytes. Patients were matched according to age and divided into groups depending on the protocol used to prevent the LH surge: the progesterone-primed group (n=118 cycles and 1,360 embryos) and the GnRH antagonist group (n=118 cycles and 1,408 embryos). Embryos were cultured in a Time-lapse imaging (TLI) incubation system. Evaluated kinetic markers were timing to pronuclei appearance (tPNa) and fading (tPNf), to two, three, four, five, six, seven, and eight cells (t2 - t8), to morulation (tM), start of blastulation (tSB) and blastulation (tB). The KID-Score ranking, durations of the second (t3-t2) and third (t5-t3) cell cycles (cc2 and cc3) and timing to complete synchronous divisions s1 (t2-tPNf), s2 (t4-t3), and s3 (t8-t5) were also calculated Embryo morphokinetics and laboratory and clinical outcomes were compared between the groups using generalized linear models, followed by the Bonferroni post hoc test, adjusted for potential confounders.**

**Results: No significant differences were noted in male or female age, maternal body mass index (BMI), total dose of FSH used for controlled ovarian stimulation, number of aspirated follicles, number of retrieved oocytes, oocyte yield, number of mature oocytes, mature oocyte rate, fertilization rate, blastocyst formation rate, or number of transferred embryos.Slower tPNa (6.2±0.2** *vs***. 7.0±0.2,** *p***=0.008), t2 (27.2±0.3** *vs***. 26.2±0.3,** *p***=0.045), t7 (56.4±** *vs***. 54.7±0.5,** *p***=0.046), tM (89.3±0.8** *vs***. 87.1±0.6,** *p***=0.045), tSB (101.5±0.8** *vs***. 110.8±0.1,** *p***=0.012) and tB (111.0±0.8** *vs***. 108.5±0.7,** *p***=0.034) were observed in embryos derived from progestin-primed cycles when than in those from the GnRH antagonist group. Significantly higher cancellation and implantation rates were observed in the progestin-primed group than in the GnRH antagonist group. However, no significant differences were noted in the pregnancy and miscarriage rates between the groups. The expense for premature ovulation prevention using a GnRH antagonist was U$ 318.18, while a total outlay of U$ 11.05 was sufficient to inhibit the premature LH surge during controlled ovarian stimulation using progestins.**

**Discussion: The selection of normal speed embryos for transfer may have led to the increased implantation rate in the progestin group, despite of the lower developmental speed. This highlights the importance of TLI for the identification and deselection of slow-growing embryos for transfer.**

**Conclusion: Exogenous progesterone replaces the use of a GnRH antagonist for prevention of premature LH surge, with the advantages of oral administration and potential cost reduction in freeze-all cycles. However, when there is no indication to freeze-all (e.g. PGT cycles or cycles at risk of ovarian hyperstimulation syndrome), the use of progestin may not be economically worthwhile and should be considered with caution.**

**O-07. Age demands to ART practice: are we arriving at new paths?**

**Maria do Carmo Borges de Souza^1^, Roberto de Azevedo Antunes^1^, Marcelo Marinho de Souza^1^, Ana Cristina Allemand Mancebo^1^, Layna Almeida Barbosa da Silva^1^, Flávia Fernandes Sequeira^1^**

^1^Fertipraxis Centro de Reprodução Humana, Rio de Janeiro, Brazil

**Introduction: The Latin America Registry by Red Latinoamericana de Reproducción Asistida (REDLARA) presents in its 2020 report that 75% of the cycles in the region are realized from women ≥35 years-old. No doubt that reproductive longevity is critical for fertility and this important scenario raises a range of challenges to societies worldwide. Healthy babies are the main priority but science behind the study of age-associated aneuploidies needs to bring us mechanisms that could be used to pathways to develop safe and effective interventions. This paper looks for checking in our daily practice the presence and the classification of aneuploidies after PGT-A tests in advanced maternal age.**

**Material and Methods: We analysed randomly 40 patients ≥35 years-old, that performed PGT-A at Fertipraxis Centro de Reprodução Humana during 2022. The objective was to detect among the embryos that arrived at the blastocyst stage, the ones with any kind of aneuploidy. There resulted 85 blastocysts that were biopsied and had the cells sent to Igenomix to perform PGT-A. After result the aneuploid embryos were classified as having simple aneuploidy, mosaic or complex aneuploidies. They were then divided into groups according to the altered chromosome based on Gruhn et al. (2019): chromosomes from 1 to 6 (group A), from 7 to 13 (group B) and from 14 to 23 (group C).**

**Results: From the 40 patients over 35 years old, only two of them had no aneuploidies at all. One was a 39 year-old woman that had three metaphase II oocytes and one euploid blastocyst. The other was 37 year-old that had 11 metaphase II and four blastocysts, all of them euploids. Both women got pregnant in the first transfer, and these pregnancies are on-going. The other 38 patients had 80 of their embryos tested as aneuploid. There were 116 chromosome errors distributed in 39 simple aneuploidies, 06 mosaics and 35 complex aneuploidies. The affected chromosomes were 18 in Group A, 26 in Group B and 72 in Group C.**

**Discussion: Natural conceptions that reach clinical recognition are 35% aneuploid. Natural fertility in humans follows an inverse U-curve, where extreme reproductive ages show reduced rates due to chromosome errors in female meiosis causing substantial pregnancy loss due to aneuploid conceptions (Gruhn et al., 2019). There is no doubt that maternal age is the only significant factor that affects aneuploidy. However, according to Gruhn et al., errors happen differently depending on women’s age. Our data agrees with them because 44% of blastocysts had complex aneuploidies (35) and 62% of the affected chromosomes were in the group C (chromosomes from 15 to 23) versus 15,5% in Group A. Even simple aneuploidies were more frequent in group C (64%). Young patients have elevated rates of Meiosis I non disjunction that affect more commonly the bigger chromosomes (1 to 5) of unclear nuclear mechanisms. Our patients are thought to present localized centromeric and cohesion weakening factors that result in the elevation in precocious separation of sister chromatids and reverse segregation.**

**Conclusion: Our data corroborate the findings that different mechanisms are responsible for the aneuploidies present in advanced maternal ages, with no solution at this moment. The challenge of integrating these newly identified causes of aneuploidy will possibly implicate in ART treatments.**

**References**

Gruhn JR, Zielinska AP, Shukla V, Blanshard R, Capalbo A, Cimadomo D, Nikiforov D, Chan AC, Newnham LJ, Vogel I, Scarica C, Krapchev M, Taylor D, Kristensen SG, Cheng J, Ernst E, Bjørn AB, Colmorn LB, Blayney M, Elder K, et al. Chromosome errors in human eggs shape natural fertility over reproductive life span. Science. 2019;365:1466-9.

Pena SDJ. Advances of aneuploidy research in the maternal germline. Nat Rev Genet. 2023;24:274.

Ruth KS, Day FR, Hussain J, Martínez-Marchal A, Aiken CE, Azad A, Thompson DJ, Knoblochova L, Abe H, Tarry-Adkins JL, Gonzalez JM, Fontanillas P, Claringbould A, Bakker OB, Sulem P, Walters RG, Terao C, Turon S, Horikoshi M, Lin K, et al. Genetic insights into biological mechanisms governing human ovarian ageing. Nature. 2021;596:393-7.

**O-08. Is the preimplantation genetic testing (PGT) associated with higher twin pregnancy rates?**

**Lilian Okada^1^, Mariangela Badalotti^1^, Marta Ribeiro Hentschke^1^, Natália Fontoura de Vasconcelos^2^, Victória Campos Dornelles^1^, Isadora Badalotti-Teloken^2^, Ricardo Azambuja^1^, João da Rosa Michelon^1^, Alvaro Petracco^1^**

^1^Fertilitat-Reproductive Medicine Center, Brazil

^2^Pontifical Catholic University of Rio Grande do Sul, Brazil

**Introduction: Preimplantation biopsy (PGT) is an assisted reproduction technique that can be used to diagnose genetic defects in IVF embryos. The PGT allows the transfer of the euploid embryos, and the technique involves the opening of the zona pellucida. It is discussed whether this method can increase the chance of twins after transfers. Thus, the objective of the present study was to compare the chance of twin pregnancy resulting from FET, with and without PGT, according to the number of embryos transferred.**

**Material and Methods: Retrospective, observational, cross-sectional study. A total of 3045 FET cycles performed between 2016 and 2021 were included. The sample was divided into 4 groups: Group 1, single embryo transfer that performed PGT (n=735); Group 2, single embryo transfer that did not perform PGT (n=1365); Group 3, transfer of 2 embryos with PGT (n=73); and Group 4, transfer of 2 embryos without PGT (n=872). The analysis compared groups 1** *vs***. 2, 3** *vs***. 4, 1** *vs***. 3, and 2** *vs***. 4. Statistical analysis was carried out with Student's t-test and Chi-square, considering significant** *p***<0.05.**

**Results: The mean maternal age between groups 1, 2, 3, and 4, respectively, was: 38.03±3.44** *vs***. 35.04±4.43** *vs***. 37.15±3.49** *vs***. 35.33±3.88,** *p***=0.000. The results found comparing groups 1** *vs***. 2, respectively, were: biochemical pregnancy (5.85%** *vs***. 5.05%,** *p***=0.000), clinical pregnancy (46.39% vs. 35.97%, p=0.000), miscarriage (10.21% vs. 20.77%, p=0.000), twins (2.63% vs. 1.01%, p=0.000). The results comparing groups 3 vs. 4 were, respectively: biochemical pregnancy (8.21% vs. 7.11%, p=0.002), clinical pregnancy (67.12% vs. 47.59%, p=0.002), miscarriage (20.4% vs. 16.86%, p=0.151), twins (26.5 vs. 19.2%, p=0.151). Evaluating the transfers of two or one embryos with PGT (group 3 vs. 1), odds ratios of 1.45 (CI 1.21-1.73) and 10.05 (CI 4.54-22.27) were observed for clinical pregnancy and twins, respectively. While the transfer of two or one embryos without PGT (group 4 vs. 2), an odds ratio of 1.62 (CI 1.36-1.92) and 22.85 (CI 9.79-64.34) was observed for clinical pregnancy and twins, respectively.**

**Discussion: Although a significant increase in twin pregnancies was observed when comparing the transfer of one euploid embryo with one embryo without PGT, there was no significant difference when comparing the transfer of two euploid embryos with two embryos without PGT. However, when considering the transfer of two euploid embryos in relation to the transfer of only one euploid embryo, the chance of pregnancy increased by 1.45, and a 10-fold increase in twinning. Considering the non-biopsied embryos, the transfer of two increased the chance of pregnancy by 1.62, and a 22-fold increase in twinning when compared to the transfer of one embryo. All embryos were transferred at the same stage, which reduced the chance of bias.**

**Conclusions: Despite the need for studies with larger samples, the results show a significant increase in twin pregnancies when comparing the transfer of one embryo with PGT versus one embryo without PGT, but not when two embryos with and without PGT were transferred. The relevant fact observed was the significant increase in the chance of twins when transferring two embryos, whether with or without PGT. These findings corroborate the need to address when planning how many embryos will be transferred, even though that although there is an increase in pregnancy rate, there is also a great increase in the risk of multiple pregnancies and all their gestational, obstetric, and perinatal consequences.**

